# Risk calculation charts for multiclass prediction models

**DOI:** 10.1186/2049-3258-73-S1-P28

**Published:** 2015-09-17

**Authors:** Vanya Van Belle, Ben Van Calster

**Affiliations:** 1 KU Leuven, Heverlee, Leuven, Belgium

## Aim

Visual representation of risk prediction models is crucial for these models to be implemented in daily clinical practice. We propose to use color charts to represent the risk estimation process of multinomial logistic regression models.

## Methods

Binary logistic regression models, where the estimated risk equals p=1/(1+exp(-b0-xb)) can be visualized by representing the contribution of each predictor (x_ib_i) with a colorbar, the color of which represents the value of the contribution. An additional colorbar is used to transform the sum of these contributions to a risk.

For multinomial models, the linear predictors (lp_l=b0^l+xb^l) are dependent on the outcome level l and a chart as described above can be made for each outcome level. For the reference level lp_ref=0 such that a chart to represent the calculation of lp_ref is not necessary. The risk on the reference level is given by p_ref=1/(1+sum_{l=1}^{k-1}exp(lp_l)), and can be visualized using k-1 colorbars, with colors encoding exp(lp_l). The conversion of the sum of these contributions exp(lp_l) to the risk can again be made by means of a monotonic transformation that can be represented in a colorbar. To visualize the risk calculation for the other outcome levels, we use the following relation: p_l=p_ref exp(lp_l), which can be written as ln(p_l)=ln(p_ref)+lp_l. Using two colorbars to represent ln(p_ref) and lp_l, the estimated risk can again be formed by means of a monotonic function of the sum of these and represented as an additional colorbar.

## Conclusion

The complete risk prediction process of multinomial regression models can be visualized by means of colorbars. Through this visualization the understanding and involvement of clinicians and patients in statistical modelling can improve. As a result, risk prediction models might be more integrated in daily clinical practice.

